# Supplementary material for: Criticality as a Determinant of Integrated Information Φ in Human Brain Networks
Source: Entropy (Basel). 2019 Oct 8;21(10):981. doi: 10.3390/e21100981 (PMC7514311; doi:10.3390/e21100981)
Supplement: Supplementary file 1 [file entropy-21-00981-s001.pdf]

Supplementary Material

Criticality as a Determinant of Integrated Information Φ in Human Brain Networks

Hyoungkyu Kim, Anthony G Hudetz, George A Mashour and UnCheol Lee*

* **Correspondence:** UnCheol Lee: uclee@med.umich.edu

1. Experimental Procedure of the Study of Sevoflurane-Induced Unconsciousness in Humans

The EEG data was recorded from seven healthy volunteers (4 males, 20–23 years of age) who gave their written informed consent participated in the study. Participants were American Society of Anesthesiologists class 1 physical status, body mass index less than 30, with Mallampati 1 or 2 airway classifications. The participants were excluded with a pregnancy, or a history of obstructive sleep apnea, gastroesophageal reflux, cardiac conduction abnormalities, asthma, epilepsy, drug use, any neurologic or psychiatric problems, and problems with anesthesia. Participants kept their eyes closed during the experiment. Sevoflurane was administered by a secured face mask at an initial concentration of 0.4% in high-flow oxygen (8 L/min). After 15-min equilibration of each concentration, the EEG data were recorded during 10-min at the target concentration. The concentration of sevoflurane was increased by levels of 0.2% until the participants lose their responsiveness(LOR). After 10-min period of LOR, the reverse protocol was proceed until the responsiveness is recovered. The participants were instructed to squeeze objects in each hand every 30 s for assessing responsiveness. 64-channels sensor cap from Electrical Geodesics, Inc. was used to record EEG with a sampling frequency 500Hz. The impedance of channels was reduced to below 50 KΩ before data acquisition.

2. Supplementary Tables

Table S1. Statistical tests of Pair Correlation Function(PCF) and integrated information($\overline{\Phi}$) among supercritical, critical, and subcritical states in model.

PCF	Supercritical	Critical	Subcritical
Supercritical		***	
Critical			***
Subcritical			

$*$ $p \leq 0.05$ $**$ $p \leq 0.01$ $***$ $p \leq 0.001$

Φ	Supercritical	Critical	Subcritical
Supercritical		***	
Critical			***
Subcritical			

$*$ $p \leq 0.05$ $**$ $p \leq 0.01$ $***$ $p \leq 0.001$

Table S2. Statistical tests of Pair Correlation Function(PCF) and integrated information($\overline{\Phi}$) among baseline, induction, anesthesia, and recovery states in experiment. The baseline, induction, anesthesia, and recovery states are the average of 10-min with 0%, 0.4%, 0.8%, 0% drug concentrations, respectively.

PCF	Baseline	Induction	Anesthesia	Recovery
Baseline			**	
Induction			*	
Anesthesia				*
Recovery				

$*$ $p \leq 0.05$ $**$ $p \leq 0.01$

Φ	Baseline	Induction	Anesthesia	Recovery
Baseline			**	
Induction			**	
Anesthesia				**
Recovery				

$*$ $p \leq 0.05$ $**$ $p \leq 0.01$

3. Supplementary Figure

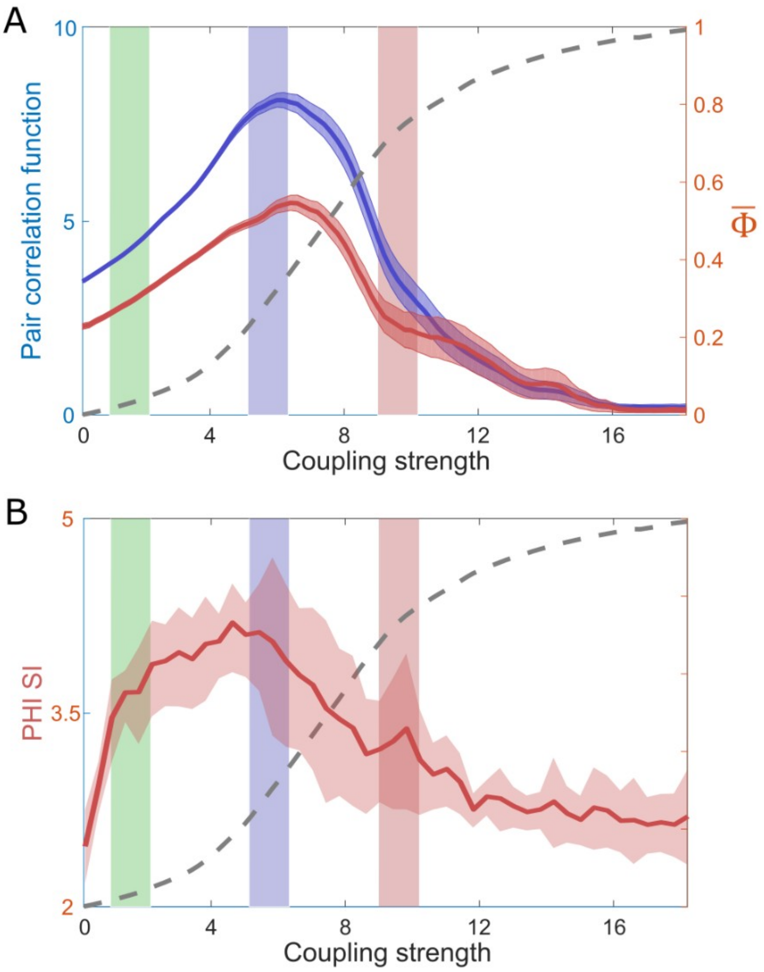

Figure S1. In order to test the robustness for the relationship between integrated information and criticality (measured by pair correlation function), we tested another integrated information measure, Φ_SI, recently introduced with its toolbox [50]. We compared the two integrated information measures, (A) $\overline{\Phi}$ in Figure 2 and (B) Φ_SI based on Queyrannes's submodular optimization algorithm [48, 50]. For Φ_SI, we iterated the simulation 10 times. The error bar indicates the standard deviation at each coupling strength. Both the integrated information measures based on different algorithms demonstrate the maximal Φ values near the critical state. Notably, we did not remove trivial partitions (e.g., partitioning the 78 nodes into 1 node and 77 nodes) in the calculation of Φ_SI, which frequenctly observed with the model data. However, for the prescise calculation of Φ_SI, it should be handled carefully in the future application.
